# Dose‐Response Efficacy and Safety of Factor XI/XIa Inhibitors in Atrial Fibrillation; a Systematic Review and Meta‐Analysis With Subgroup Exploration and Trial Sequential Validation

**DOI:** 10.1002/clc.70263

**Published:** 2026-02-12

**Authors:** Muhammad Aqib Faizan, Tooba Rehman, Mrunalini Dandamudi, Jaivardhan A. Menon, Victoria Zecchin Ferrara, Zeyad Kholeif, Alina Tanvir, Fatima Saeed, Jibran Ikram, Moiuz Chaudhri, Carlos Espiche, Main Muhammad Salman Aslam, Zainab Humayun, Ahmad Mustafa Khalid, Saad Ahmad Waqas, Raheel Ahmad, Luis Cerna

**Affiliations:** ^1^ Gomal Medical College Khyber Medical University Peshawar Pakistan; ^2^ Montefiore Einstein Medical Center Bronx New York USA; ^3^ Division of Pulmonary and Critical Care Medicine, Department of Medicine Brigham and Women's Hospital Boston Massachusetts USA; ^4^ Harvard Medical School Boston Massachusetts USA; ^5^ Faculty of Medicine and Surgery University of Padua Padua Italy; ^6^ Cardiovascular department Mayo Clinic Rochester Minnesota USA; ^7^ King Edward Medical University Lahore Pakistan; ^8^ Massachusetts General Hospital Boston USA; ^9^ Cardiovascular Medicine Department Heart, Vascular and Thoracic Institute, Cleveland Clinic Cleveland Ohio USA; ^10^ Hackensack Meridian Ocean University Medical Center Brick New Jersey USA; ^11^ Department of Pulmonary and Critical Care Medicine University of Michigan Ann Arbor Michigan USA; ^12^ University of Missouri‐Kansas City Missouri USA; ^13^ Abbottabad International Medical College Abbottabad Pakistan; ^14^ Dow University of Health Sciences Karachi Pakistan; ^15^ Imperial College London London UK; ^16^ George Washington University Hospital Washington DC USA

**Keywords:** anticoagulation, atrial fibrillation, direct oral anticoagulants, factor XI/XIa

## Abstract

**Background:**

Factor XI/XIa inhibitors are emerging anticoagulants with potential to reduce bleeding complications in atrial fibrillation (AF) patients. This meta‐analysis evaluated their efficacy and safety compared to direct oral anticoagulants (DOACs) and explored dose optimization.

**Methods:**

A systematic search of PubMed, Cochrane, and Embase was conducted through March 2025 following PRISMA guidelines. Randomized controlled trials (RCTs) comparing Factor XI/XIa inhibitors with DOACs in AF patients were included. Outcomes assessed were major bleeding, stroke, systemic embolism, all‐cause and cardiovascular mortality and serious adverse events. Risk ratios (RR) with 95% confidence intervals (CI) were pooled using a Mantel‐Haenszel random‐effects model. Heterogeneity was evaluated with the I² statistic, and evidence certainty assessed by the GRADE approach. Trial Sequential Analysis (TSA) was performed.

**Results:**

Three RCTs including 16,772 patients (mean age 73 years, CHA₂DS₂‐VASc 3.9–5) were analyzed. Factor XI/XIa inhibitors significantly reduced major bleeding (RR: 0.41, 95% CI: 0.36–0.46, I² = 0%) compared to DOACs. However, stroke risk was increased (RR: 3.42, 95% CI: 2.62–4.46), particularly with asundexian 50 mg (RR: 4.02). No significant differences were observed in all‐cause mortality (RR: 0.82) or cardiovascular death (RR: 1.05). Systemic embolism risk was higher (RR: 4.26), while serious adverse events were comparable (RR: 0.95). TSA indicated encouraging safety outcomes but highlighted the need for further large‐scale studies.

**Conclusion:**

Factor XI/XIa inhibitors lower major bleeding risk in AF patients but increase stroke and systemic embolism rates without impacting mortality.

## Graphical abstract

Efficacy and Safety of Factor XI/XIa Inhibitors vs DOAC in Atrial Fibrillation.

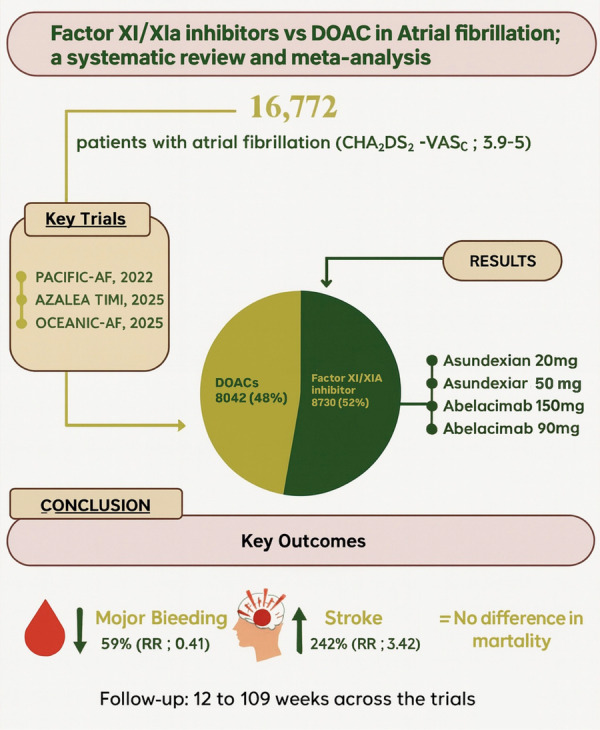

## 1 Introduction

Atrial fibrillation (AF) affects more than fifty million worldwide and presents as a considerable clinical and societal challenge [1, 2, 3]. It has been linked to higher risk of death and thromboembolic events, particularly stroke [3, 4, 5]. Previous guidelines focused on vitamin K antagonists for stroke prevention in AF, with little emphasis on the non‐vitamin K antagonists NOACs (also referred to as direct oral anticoagulants DOACs) like rivaroxaban and apixaban. However, due to the evidence from the recent clinical trials, both European and North American guidelines suggest DOACs as the first‐line agents for ischemic stroke prevention in AF. This shift towards DOACs is driven by their improved risk benefit profile as compared to traditional anticoagulants, particularly in reducing major and intracranial bleeding risks, though gastrointestinal bleeding remains a persistent concern [6, 7, 8, 9]. In order to address these concerns, the coagulation XI pathway is the focus of recent research.

FXI is a key component of the intrinsic pathway of coagulation; however, it is considered nonessential for physiological hemostasis [10, 11]. Emerging evidence from studies on factor XI/XIa inhibitors has shown their potential as safer alternatives for anticoagulation therapy. Among these, asundexian and abelacimab have emerged as novel agents, demonstrating a favorable profile in terms of safety as compared to current ongoing treatment therapies [12, 13, 14].

Despite the growing use of factor XI/XIa inhibitors for treatment in AF, a significant gap in the literature persists regarding the impact of varying dosages on both efficacy and safety. Previous studies, though valuable, have been limited by small sample sizes and inconsistent focus on dose‐response relationships [15, 16, 17]. This has led to inconclusive findings on the optimal dosing strategy for maximizing therapeutic benefits while minimizing adverse effects such as major clinical bleeding. The lack of a comprehensive meta‐analysis on factor XI/XIa inhibitors dose variability exacerbates these uncertainties, leaving clinicians with limited guidance on how to optimize treatment. By synthesizing data from multiple studies, this meta‐analysis aims to resolve these inconsistencies, providing a robust evaluation of factor XI/XIa inhibitors efficacy and safety across different doses. Ultimately, this work seeks to offer a practical solution to the real‐world challenge of dose optimization, equipping clinicians with evidence‐based insights to improve patient outcomes in AF management. Furthermore, the trial sequential analysis (TSA) performed in this study would provide valuable insights into the current evidence base surrounding Factor XI/Xla inhibitors and the need for further adequately powered trials to confirm the beneficial use of these elements in atrial fibrillation patients.

## 2 Methods

This systematic review and meta‐analysis was conducted and reported in accordance with Cochrane Collaboration Handbook for Systematic Reviews of Interventions and the Preferred Reporting Items for Systematic Reviews and Meta‐Analysis (PRISMA) Statement guidelines [18]. The protocol was registered in the International Prospective Register of Systematic Reviews (PROSPERO) under record number 1018490.

### 2.1 Data Source and Search Strategy

We systematically searched PubMed, Cochrane, and Embase databases for studies published from database inception to March 2025. The entire search strategy is given in Supplemental table 1. Two authors (MAF and TR) independently evaluated full‐text publications for inclusion based on predetermined criteria after eliminating duplicates and screening titles and abstracts. A panel discussion with the third author (MD) settled the disagreements.

**Figure 1:**
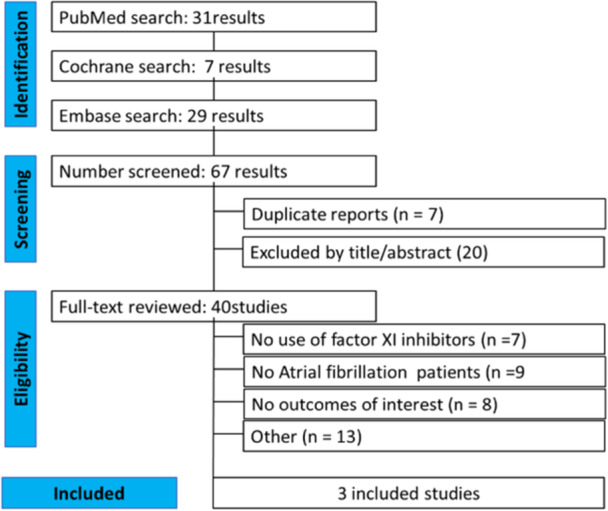
PRISMA flow diagram showing the search strategy and study selection process for meta‐analysis.

Moreover, we searched for additional eligible studies by reviewing the references from the included studies. The PRISMA flowchart, illustrating the search results, can be found in Figure 1.

### 2.2 Eligibility Criteria

Studies that fulfilled the following eligibility criteria were included: (1) Randomized control studies; (2) enrolling patients with Atrial fibrillation; (3) comparing patients with different doses of factor XI/XIa inhibitors versus patients with DOAC; and (4) reporting any of the endpoints of interest that have been predetermined.

Exclusion criteria were: (1) absence of a control group; (2) patients without atrial fibrillation; (3) studies enrolling ≤ 10 patients; (4) conference abstracts or (5) studies with overlapping populations.

### 2.3 Data Extraction

Data extraction was done independently by two writers (MAF and TR), and disagreements were settled in a group discussion with the third author (M.D.). Using a standard data extraction form, the first author of the studies, the publication year, sample size, patient characteristics (age, sex ratio), intervention details, and clinical outcomes were all included in the data extracted from the eligible studies. For dichotomous outcomes, we retrieved the total number of patients and the number of incidents for each outcome. Baseline characteristics were reported as the mean ± standard deviation for continuous variables and totals along with percentages for binary variables.

### 2.4 Study Endpoints and Subgroup Analyses

Outcomes of interest included Stroke prevention, bleeding risk (major, minor, intracranial), all‐cause mortality, thromboembolic events.

We performed subgroup analysis based on various doses of the intervention drug to determine the efficacy and safety of the drug across different doses. Heterogeneity was assessed using *I²* statistics, and a two‐tailed *p*‐value of < 0.05 indicates statistical significance.

### 2.5 Statistical Analysis

Review Manager (RevMan, version 5.4; The Cochrane Collaboration, Copenhagen, Denmark) was used to do the statistical analysis. It calculated the risk ratios (RR) for dichotomous outcomes along with the corresponding 95% Confidence Intervals (CIs). A random‐effects model Mantel‐Haenszel method was employed to address heterogeneity among studies. The pooled results were represented graphically as forest plots. The restricted maximum likelihood estimator was used to calculate heterogeneity variance tau². Heterogeneity was assessed with Cochrane's *Q* statistic and Higgins and Thompson's I² statistic [19]. The consistency of the studies was determined based on I² values of 0%, ≤ 25%, ≤ 50%, and > 50%, indicating no observed, low, moderate, and substantial heterogeneity, respectively. Statistical significance was indicated by *p*‐values < 0.05.

### 2.6 Trial Sequential Analysis (TSA)

Trial sequential analysis was carried out through Trial Sequential Analysis software version 0.9.5.10 beta [Computer program] (The Copenhagen Trial Unit, Centre for Clinical Intervention Research, The Capital Region, Copenhagen University Hospital‐Rigshospitalet). We conducted TSA and evaluated the adequacy of the available patient population to determine the implications for future research. We determined the necessary information size adjusted for diversity. The sum of variability between trials and a sampling error estimate based on the necessary information size is used to compute diversity, which shows the percentage of variability between trials. We performed TSA with the goal of maintaining an overall 5% rise or type I error, which is considered the standard for most meta‐analyses and systematic reviews.

### 2.7 Grade Certainty Assessment

The certainty of evidence for each outcome was evaluated using the GRADE (Grading of Recommendations Assessment, Development and Evaluation) framework. We employed the GRADEpro GDT software to assess evidence quality across five key domains.

A Summary of Findings (SoF) table was generated to display relative and absolute effects, participant numbers, and the overall confidence in the estimates.

### 2.8 Quality Assessment

Quality of the included randomized controlled trials in meta‐analysis was evaluated by using Risk of Bias 2 (RoB‐2) tool [20]. Each study was assessed in 5 domains: randomization, deviation from intended intervention, due to missing outcome data, measurement of outcome and selection of the reported results.

## 3 Results

### 3.1 Search Results and Study Characteristics

In this meta‐analysis of selected randomized controlled trials (PACIFIC‐AF 2022 [12], AZALEA TIMI 2025 [13], and OCEANIC‐AF 2025 [14]), a total of 16,772 participants were included. Of these, 8,730 participants randomised to Factor XI/XIa inhibitors group were compared with those of 8,042 participants recieving direct oral anticoagulant across multiple clinical outcomes. Table 1 provides specifics on the research features of the randomised control trials that were part of the meta‐analysis. The mean age of the study population was 74 years, with 6,095 (36.1%) being female. The CHA₂DS₂‐VASc score was 3.9 to 5. PACIFIC‐AF 2022 [12] (*n* = 755) and OCEANIC‐AF 2025 [14] (*n* = 14,810) assessed asundexian (20 mg or 50 mg) compared with apixaban (5 mg twice daily), with follow‐up periods of 12 and 46 weeks, respectively. AZALEA TIMI 2025 [13] (*n* = 1,287) compared abelacimab (90 mg or 150 mg) with rivaroxaban (20 mg) over a follow‐up period of 109 weeks. Notably, this study had a higher proportion of female participants and a greater mean CHA₂DS₂‐VASc score compared to the others. Reporting bleeding risk was carried out differently in each study. PACIFIC‐AF 2022 [12] included patients who had a history of bleeding that required medical attention during the previous 12 months, at least one bleeding risk factor, an estimated glomerular filtration rate (eGFR) of 30–50 mL/min, or a current aspirin prescription. AZALEA TIMI 2025 [13] reported 50.3% of patients with a HAS‐BLED score > 3.5 but did not stratify them into specific risk groups. Although, OCEANIC‐AF 2025 [14] lacked baseline bleeding risk data, it includes patients who met identical bleeding risk criteria as PACIFIC‐AF 2022 [12].

**Table 1:** Study characteristics of the included randomized controlled trials.

Study	Total number of patients (n)	Drug dosage (mg)	Number	Age (years)	Femalen (%)	Diabetesn (%)	Hypertensionn (%)	Previous stroken (%)	DOACn (%)	CHA2DS2‐VASc score	Follow up
PACIFIC‐AF 2022	755	Asundexian 20 mg once daily	251	73.6 (8.0)	103 (41)	83 (33)	226 (90)	22 (9)	109 (43)	3.9 (1.4)	12 weeks
		Asundexian 50 mg once daily	254	71.1 (8.5)	97 (38)	74 (29)	227 (89)	18 (7)	116 (46)	3.8 (1.3)	12 weeks
		Apixaban 5 mg twice daily	250	74.3 (8.3)	109 (44)	87 (35)	220 (88)	25 (10)	111 (44)	4.1 (1.4)	12 weeks
AZALEA TIMI 2025	1287	Abelacimab 150 mg SQ once monthly	430	74 (6.7)	194 (44.9)	231 (53.7)	417 (97.0)	59 (13.7)	271 (63.0)	5.0 (4.0–5.0)	109 weeks
		Abelacimab 90 mg SQ once monthly	427	75 (7.4)	195 (45.7)	223 (52.2)	410 (96.0)	38 (8.9)	283 (66.4)	5.0 (4.0–5.0)	109 weeks
		Rivaroxaban 20 mg once daily	430	74 (7.4)	184 (42.8)	245 (57.0)	418 (97.2)	75 (17.5)	291 (57.7)	5 (4.0–6.0)	109 weeks
OCEANIC‐AF2025	14810	Asundexian 50 mg once daily	7415	73.9 (7.7)	2656 (35.8)	2722 (36.7)	6558 (88.4)	1389 (18.7)	1238 (16.7)	4.3 + –1.3	46 weeks
		Apixaban	7395	73.9 (7.7)	2558 (36.6)	2748 (37.2)	6565 (88.8)	1305 (17.6)	1255 (17.0)	4.3 + –1.3	46 weeks

DOAC: Direct Oral Anticoagulant, CHA2DS2‐VASc score: Cardiac failure, Hypertension, Age ≥ 75 years, Diabetes, Stroke/TIA/thromboembolism, Vascular disease, Age 65–74 years, Sex category (female sex).

### 3.2 Pooled Results

#### 3.2.1 Major Bleeding

**Figure 2:**
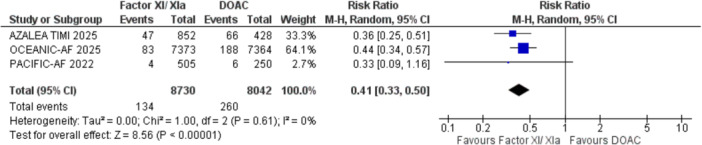
Forest plot for the outcome of major bleeding; it shows the result of pooled analysis comparing the effect of factor XI/XIa inhibitor treatment to the control group on major bleeding. CI, confidence interval; DOAC, direct oral anti‐coagulants; IV, inverse variance; RR, relative risk.

Factor XI/XIa inhibitors demonstrated a significantly lower risk of major bleeding as compared to DOAC (RR: 0.41, 95% CI: 0.36–0.46; Figure 2) with no significant heterogeneity (Tau² = 0.00, I² = 0%) and subgroup analyses for specific doses (Asundexian 20 mg and 50 mg, Abelacimab 150 mg, and 90 mg) showed risk ratios ranging from 0.32 to 0.50, indicating a consistent reduction in major bleeding across all subgroups (supplementary figure 1).

#### 3.2.2 Ischemic Stroke

**Figure 3:**
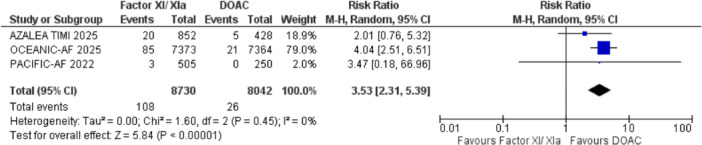
Forest plot for the outcomes of ischemic stroke; it shows the result of pooled analysis comparing the effect of factor XI/XIa inhibitor treatment to the control group regarding ischemic stroke. CI, confidence interval; DOAC, direct oral anti‐coagulants; IV, inverse variance; RR, relative risk.

A significantly higher incidence of ischemic stroke was observed in the experimental group, with overall (RR: 3.42, 95% CI: 2.62‐4.46, I² = 0%, *p* < 0.0001; Figure 3). In the subgroup analysis, Asundexian 50 mg had the most consistent and significant increase in risk (RR = 4.02). In contrast, Asundexian 20 mg and Abelacimab (both 150 mg and 90 mg) didn't reveal any difference than the DOAC (*p* > 0.05) for the outcome of ischemic stroke (supplementary figure 2).

#### 3.2.3 Hemorrhagic Stroke

**Figure 4:**
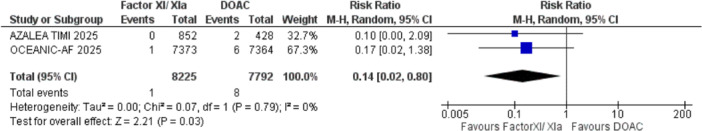
Forest plot for the outcome of hemorrhagic stroke; it shows the result of pooled analysis comparing the effect of factor XI/XIa inhibitor treatment to the control group regarding hemorrhagic stroke. CI, confidence interval; DOAC, direct oral anti‐coagulants; IV, inverse variance; RR, relative risk.

In contrast to ischemic stroke, there was no difference between factor XI/XIa inhibitors and direct oral anticoagulants regarding the risk of hemorrhagic stroke. (RR: 0.14, 95% CI: 0.01‐2.85, I^2^ = 0%, *p* = 0.08, Figure 4).

#### 3.2.4 Systemic Embolism

**Figure 5:**
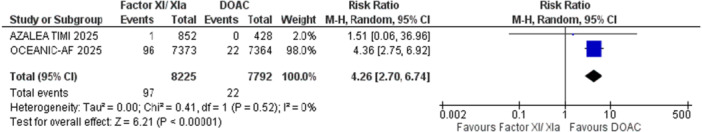
Forest plot for the outcome of systemic embolism; it shows the result of pooled analysis comparing the effect of factor XI/XIa inhibitor treatment to the control group on systemic embolism. CI, confidence interval; DOAC, direct oral anti‐coagulants; IV, inverse variance; RR, relative risk.

The risk of systemic embolism was also significantly higher (RR: 4.26, 95% CI: 0.63‐28.73; Figure 5) in Factor XI inhibitors treatment group in comparison with direct oral anticoagulants.

#### 3.2.5 Mortality

**Figure 6:**
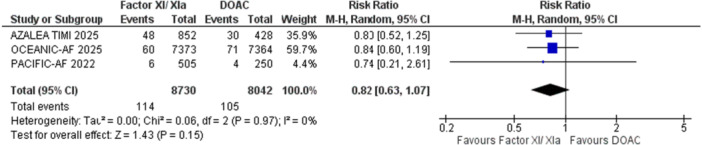
Forest plot for the outcome of all‐cause mortality; it shows the result of pooled analysis comparing the effect of factor XI/XIa inhibitor treatment to DOAC on all‐cause mortality. CI, confidence interval; DOAC, direct oral anti‐coagulants; IV, inverse variance; RR, relative risk.

**Figure 7:**
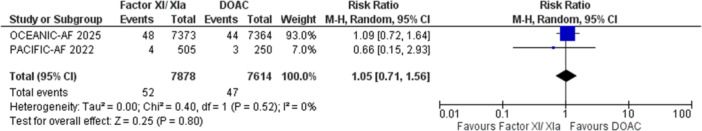
Forest plot for the outcome of cardiovascular mortality; it shows the result of pooled analysis comparing the effect of factor XI/XIa inhibitor treatment to DOAC on cardiovascular mortality. CI, confidence interval; DOAC, direct oral anti‐coagulants; IV, inverse variance; RR, relative risk.

Factor XI inhibitors exhibited no significant difference in all‐cause mortality (RR: 0.82, 95% CI 0.77‐0.88, *p* = 0.80; Figure 6) or cardiovascular death (RR: 1.05, 95% CI 0.21‐5.33, *p* = 0.11; Figure 7) when compared to DOACs. Similarly, subgroup analyses for specific doses of different factorXI/Xia inhibitors (Asundexian 50 mg, Abelacimab 150 mg, and 90 mg) didn't reveal any significant difference in mortality (*p* > 0.05) than DOAC (supplementary figure 3).

**Figure 8:**
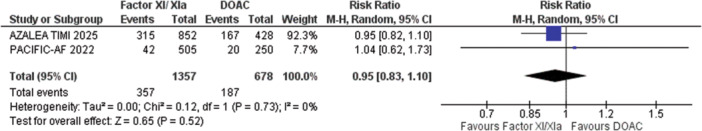
Forest plot for the outcome of serious adverse events; it shows result of pooled analysis comparing the effect of factor XI/XIa inhibitor treatment to the control group on the incidence of serious adverse events. CI, confidence interval; DOAC, direct oral anti‐coagulants; IV, inverse variance; RR, relative risk.

**Serious adverse events**; that encompass cardiac events like atrial fibrillation, cardiac failure, myocardial infarction, or gastrointestinal events or hepatobiliary disorder, showed no significant difference (RR: 0.95, 95% CI: 0.70‐1.31, *p* = 0.12 Figure 8) between factor XI/Xia and DOAC. All analyses exhibited low heterogeneity (I² = 0%), suggesting consistent effects across studies and doses.

### 3.3 GRADE Assessment

According to the GRADE evaluation as detailed in Summary of Finding table, Supplemental table 2, there was high‐certainty evidence indicating that Factor XI/XIa inhibitors are associated with a reduced risk of both all‐cause mortality and major bleeding when compared to DOACs. In contrast, the likelihood of ischemic stroke was higher with Factor XI/XIa inhibitors, also supported by high‐certainty evidence. The data for hemorrhagic stroke and systemic embolism were rated as low certainty due to substantial imprecision and limited event numbers. Certainty for cardiac death and serious adverse events was graded as moderate, mainly because of wide confidence intervals.

### 3.4 Trial Sequential Analysis

In this meta‐analysis, we performed Trial Sequential Analysis (TSA) to better understand how reliable and conclusive the current evidence is on Factor XI/XIa inhibitors compared to direct oral anticoagulants (DOACs). The cumulative Z‐curves for most outcomes did not cross the Trial Sequential Analysis (TSA) boundaries for statistical significance, indicating that the available evidence remains insufficient to confirm or reject a clinical effect, and thus further studies are required. However, the consistent directionality of the data across outcomes—particularly for major bleeding and mortality—is suggestive of benefit from Factor XI/XIa inhibitors.

#### 3.4.1 Major Bleeding

Our cumulative meta‐analysis demonstrated a consistent trend toward a lower incidence of major bleeding events with Factor XI/XIa inhibitors. Although these results did not reach conventional statistical thresholds for definitive conclusion, the direction of effect is clinically promising (Supplemental Figure 4A). This observation suggests that Factor XI/XIa inhibitors may confer a more favorable bleeding safety profile compared with Direct Oral Anticoagulants (DOACs), an outcome that warrants validation in future randomized controlled trials. Trial Sequential Analysis (TSA) reinforces this interpretation; while the cumulative Z‐curve did not cross the TSA monitoring boundary, the consistent direction of effect indicates a potential clinical benefit. TSA thus underscores the necessity for additional, adequately well‐powered, event‐driven randomized trials to confirm these findings.

#### 3.4.2 Ischemic Stroke

In the analysis of ischemic stroke, the cumulative Z‐curve remained within the inner futility region and did not cross either the superiority or futility boundaries (Approximate RIS 2,886). Currently, there is no statistically robust evidence to support either a clinical benefit or harm associated with Factor XI/XIa inhibition for this outcome. Nonetheless, the available data suggest that these agents are at least comparable to DOACs in the prevention of ischemic stroke, with no emergent safety concerns. From a TSA perspective, these findings reflect insufficient power for conclusive results, but the point estimate trend remains reassuring. This distinction between statistical insignificance and clinical stability is important, and further investigation is required to solidify the evidence base.

#### 3.4.3 Systemic Embolism

For systemic embolism, the meta‐analysis results were statistically neutral. The pooled data neither confirm nor exclude a treatment benefit but, importantly, show no indication of increased risk (Supplemental Figure 4C). While the current cumulative sample size remains below the required information size for a conclusive result, TSA reveals that the Z‐curve has not crossed any significance boundaries. This highlights the need for further research while supporting the preliminary view that Factor XI/XIa inhibitors perform similarly to DOACs in this outcome domain.

#### 3.4.4 Hemorrhagic Stroke

Despite the inclusion of a relatively large cumulative cohort, the analysis remains underpowered to draw definitive conclusions regarding the risk of hemorrhagic stroke. The cumulative Z‐curve is centered and has not crossed any significance boundaries (Supplemental Figure 4D). No concerning safety signals have emerged. TSA confirms the inconclusive nature of the current evidence and underscores the importance of continued investigation in this critical area, particularly in patient cohorts at high risk for intracranial hemorrhage.

#### 3.4.5 All‐Cause Mortality

The analysis of all‐cause mortality revealed a trend toward reduced mortality with Factor XI/XIa inhibitors. Although statistical significance was not achieved, the consistent direction of the effect across studies is encouraging and supports further investigation (Supplemental Figure 4E). TSA results reinforce this interpretation; the Z‐curve did not cross the significance boundary, but the pattern suggests a possible clinical benefit. These preliminary findings highlight the potential promise of Factor XI/XIa inhibition in improving survival, pending confirmation from adequately powered randomized controlled trials.

#### 3.4.6 Cardiovascular Mortality

The pattern observed for cardiovascular mortality closely mirrored that of all‐cause mortality (Supplemental Figure 4F). While definitive conclusions cannot yet be drawn due to insufficient power, the consistent trend toward benefit is noteworthy. TSA did not indicate statistical significance, yet the observed directionality supports continued exploration of potential improvements in cardiovascular outcomes through Factor XI/XIa inhibition.

#### 3.4.7 Serious Adverse Events

Regarding serious adverse events, the current cumulative data remain insufficient to make firm conclusions. Crucially, no alarming safety signals have been identified thus far. TSA findings are consistent with this interpretation, indicating that the cumulative evidence is underpowered but overall stable (supplemental Figure 4 (G). As with other outcomes, ongoing and future trials will be critical to more precisely delineate the complete safety profile of Factor XI/XIa inhibitors.

### 3.5 Risk of Bias Assessment

The risk of bias assessment, conducted using the RoB 2 tool [20], is displayed as supplemental Figure 5 and supplemental Figure 6. It highlights some concerns in multiple domains across the included studies. Bias arising from the randomization process was generally rated as low risk across most studies. Furthermore, bias in measurement of the outcome was not found. However, Bias in the selection of the reported result and missing outcome data were found to be concerning, with a considerable percentage of studies displaying some level of concern. The overall risk of bias was judged as “some concerns”, primarily due to missing outcome data and the selection of reported results. These findings suggest that, while the included trials maintain a generally robust methodology, potential limitations in outcome reporting and follow‐up completeness should be considered when interpreting the results.

## 4 Discussion

This meta‐analysis evaluates the efficacy and safety of Factor XI/XIa inhibitors compared to direct oral anticoagulants (DOACs) in patients with atrial fibrillation, based on data from the PACIFIC‐AF 2022, AZALEA TIMI 2025, and OCEANIC‐AF 2025 trials, encompassing a total of 16,772 participants. Our key finding is the significantly lower risk of major bleeding associated with Factor XI/XIa inhibitors when compared to DOACs. The overall risk ratio suggested a consistent reduction in bleeding with no heterogeneity across all the analyzed studies. This finding highlights a key advantage of using Factor XI/XIa inhibitors in clinical practice and aligns with existing research suggesting that Factor XI/XIa inhibitors offer a promising approach to maintain anticoagulation efficiently in patients who have a high risk of bleeding [15, 16, 21]. Furthermore, apixaban is known in the literature as the anticoagulant having the least risk of related bleeding [22, 23], FXIa inhibitors’ comparative advantage over Apixaban further validates its capacity to reduce bleeding risk and safeguard high‐risk patients.

Our subgroup analyses for specific doses of Asundexian and Abelacimab yielded risk ratios ranging from 0.32 to 0.50, further supporting this favorable bleeding profile; suggesting that Factor XI/XIa inhibitors may offer a safer anticoagulation alternative for patients at high risk of hemorrhagic complications across all its dosages.

We observed no significant difference in all‐cause mortality or cardiovascular death between Factor XI/XIa inhibitors and DOACs. Subgroup analyses across different doses of Asundexian (50 mg) and Abelacimab (150 mg, 90 mg) suggest consistent mortality risk ratios ranging from 0.50 to 0.87, with no significant heterogeneity, reinforcing the robustness of these findings as well. Previous studies have suggested that Factor XI/XIa inhibitors may achieve effective anticoagulation while minimizing thrombotic risk, though these results indicate that they do not significantly alter mortality rates [24].

A significantly higher incidence of stroke was observed with Factor XI/XIa inhibitors in our analysis. Prior relevant research demonstrated either no clarity on the interventional impact of FXI inhibition on stroke outcomes [15], or increased risk with Factor XI/Xia inhibitors [16, 17]; findings consistent with our results. Notably, Asundexian 50 mg exhibited the highest and most consistent increase in stroke risk (RR = 4.02), whereas Asundexian 20 mg had less certainty due to a wide confidence interval. Abelacimab (150 mg and 90 mg) also showed an approximate twofold increased risk of stroke (RR ≈ 2.00), but the confidence intervals crossed 1, indicating a lack of statistical significance at these doses. These findings align with concerns raised in prior research suggesting that the thrombotic protection conferred by Factor XI/XIa inhibitors may not be as robust as that provided by DOACs, particularly in patients with high thromboembolic risk [25]. Although AF is well recognized to confer a risk of stroke, this risk is not homogeneous. The mean CHA₂DS₂‐VASc score across all studies were 3.9 to 5 indicating that the patient cohort was at a higher risk of stroke in both treatment and control groups of these studies. Current AHA guidelines for managing patients with AF strongly support anticoagulation for patients with a CHA₂DS₂‐VASc score of ≥ 2 in men and ≥ 3 in women [2]. Our observations lead us to believe that while the newer generation Factor XI/XIa inhibitors provide efficacious and clinically superior anticoagulation, they may not be suitable for patients with a high stroke risk stratification score.

Despite these concerns, serious adverse events did not significantly differ between Factor XI inhibitors and DOACs suggesting overall a good tolerability. This highlights that while the safety profile in terms of adverse events remains comparable, the elevated stroke risk necessitates a cautious approach to Factor XI/XIa inhibitor use, especially in patients with a high stroke risk stratification score.

The risk of bias assessment, conducted using the RoB 2 tool, indicated some concerns, particularly in missing outcome data and selection bias in reporting results. While the trials maintained generally robust methodologies, these potential limitations should be considered when interpreting the findings. The lack of standardized bleeding risk assessment across trials also introduces variability in the reported safety outcomes, which may influence clinical decision‐making.

In our analysis, the cumulative Z‐curves for most outcomes did not cross the Trial Sequential Analysis (TSA) boundaries for statistical significance, indicating that the available evidence remains insufficient to confirm or reject a clinical effect, and thus further studies are required. However, the consistent directionality of the data across outcomes—particularly for major bleeding and mortality—is suggestive of benefit from Factor XI/XIa inhibitors. It is important to distinguish between statistical and clinical significance in interpreting TSA findings. Although the statistical thresholds have not been met, the consistent trends in the point estimates across some of the endpoints suggest potential clinical benefit. TSA thus underscores the need for additional, well‐powered, event‐driven randomized trials to confirm these initial signals and clarify the full clinical role of these novel agents.

While Factor XI/Xia inhibitors reflect a promising treatment strategy, their appropriate clinical role requires additional exploration. Current evidence, derived from trials enrolling high‐thromboembolic‐risk patients (mean CHA2DS2‐VASc scores 3.9‐5.0), suggests a potential safety advantage in bleeding reduction. This profile may be particularly beneficial for high‐risk patients in whom bleeding concerns limit anticoagulation. Yet, the noticeable increase in thromboembolic events highlights the need for careful interpretation and longer‐term data to accurately assess net therapeutic benefit. Additionally, in order to ascertain if Factor Xl/Xia inhibition can fulfill its promise of uncoupling anticoagulation from hemostasis across the board, head‐to‐head comparisons with typical DOACs and data from developing agents within the class (such as abelacimab and asundexian) will be crucial across the spectrum of atrial fibrillation. Furthermore, mechanistic studies exploring the impact of Factor XI inhibition on thrombus stability and clot propagation may provide insights into mitigating the increased stroke risk observed in this meta‐analysis.

### 4.1 Limitations

This systematic review and meta‐analysis has some limitations that must be acknowledged. First, only three RCTs were included which may affect the overall statistical power and the generalizability of findings. Second, the variations in sample sizes, ethnicities and baseline characteristics could contribute to clinical heterogeneity and may have an influence on pooled results. Furthermore, the consistency of the results might have been affected by the difference in follow up durations across the studies. For instance, the follow up duration of PACIFIC‐AF and OCEANIC‐AF trial were 12 and 46 weeks, respectively, while 46 weeks for AZALEA TIMI trial. Third, the trials included in our analysis are comprised of patients with relatively high CHA₂DS₂‐VASc scores (3.9–5), limiting the applicability of these findings to lower‐risk AF populations. Fourth, although the risk of bias assessment indicated a generally robust methodology, there were some concerns regarding missing outcome data and selective outcome reporting, which could introduce reporting bias.

## 5 Conclusion

This meta‐analysis of randomized controlled trails, evaluating the efficacy and safety of various doses of factor XI/XIa inhibitors compared with DOACs in patients with atrial fibrillation demonstrates a strong safety profile in terms of significantly lower risk of major bleeding with consistent results across various dose subgroups and no heterogeneity. However, their use can lead to a markedly higher risk of ischemic stroke, particularly if receiving 50 mg of Asundexian. This highlight concerns regarding thromboembolic events in high‐risk individuals (mean CHA_2_DS_2_‐VASc 3.9‐5). Additionally, there was not a significant difference in cardiovascular death, all‐cause mortality, or major adverse events while using Factor XI/XIa inhibitors, indicating general tolerance and neutrality in survival outcomes.

## Funding

The authors received no specific funding for this work.

## Disclosures

All authors report no relationships that could be construed as a conflict of interest. All authors take responsibility for all aspects of the reliability and freedom from bias of the data presented and their discussed interpretation.

## Conflicts of Interest

The authors declare no conflicts of interest.

## Supporting information


**Supplemental Figure 1:** Dose‐based subgroup analysis for the outcome of major bleeding. **Supplemental Figure 2:** Dose‐based subgroup analysis for the outcome of ischemic stroke. **Supplemental Figure 3:** Dose‐based subgroup analysis for the outcome of all‐cause mortality. **Supplemental Figure 4:** Trial Sequential Analysis (TSA) assessing the effect of Factor XI/XIa inhibitors verses DOAC on the outcomes of (A) Major Bleeding (B) Ischemic stroke (C) Haemorrhagic stroke (D) Systemic embolism (E) All‐cause mortality (E) Cardiovascular mortality (F) Serious Adverse Events. **Supplemental Figure 5:** Risk of bias summary. **Supplemental Figure 6:** Risk of bias graph. **Supplemental Table 1:** Detailed search strategy across different data bases. **Supplemental Table 2:** Summary of Findings Table (GRADE Assessment).

## Data Availability

The data that support the findings of this study are openly available in https://pubmed.ncbi.nlm.nih.gov/ at https://pubmed.ncbi.nlm.nih.gov/, reference number https://pubmed.ncbi.nlm.nih.gov/.
