# Supplementary material for: Dose‐Response Efficacy and Safety of Factor XI/XIa Inhibitors in Atrial Fibrillation; a Systematic Review and Meta‐Analysis With Subgroup Exploration and Trial Sequential Validation
Source: Clin Cardiol. 2026 Feb 12;49(2):e70263. doi: 10.1002/clc.70263 (PMC12895463; doi:10.1002/clc.70263)

**Supplemental table1**.Detailed search strategy across different data bases

| Database | Search Strategy | Results |
| --- | --- | --- |
| PubMed | (“Factor XI inhibitor" OR "Factor XIa inhibitor" OR "Abelacimab" OR "Milvexian" OR "Asundexian" OR "Osocimab") AND ("atrial fibrillation" OR AF OR"Atrial Fibrillation"[Mesh] OR "non-valvular atrial fibrillation" OR NVAF) AND prevention AND (stroke OR thromboembolism OR "Stroke"[Mesh]) AND ("anticoagulation" OR "anticoagulant" OR "Anticoagulants"[Mesh] OR DOAC OR NOAC OR warfarin OR "Warfarin"[Mesh] OR "vitamin K antagonist" OR VKA) | 31 |
| Cochrane | "Factor XI inhibitor" OR "Factor XIa inhibitor" OR "Abelacimab" OR "Milvexian" OR "Asundexian" OR "Osocimab") AND ("atrial fibrillation" OR "AF" OR "non-valvular atrial fibrillation" OR "NVAF") AND ("stroke prevention" OR "thromboembolism prevention" OR "cardioembolic stroke") AND ("anticoagulation" OR "anticoagulant" OR "direct oral anticoagulants" OR "DOAC" OR "NOAC" OR "warfarin" OR "vitamin K antagonist" OR "VKA") | 7 |
| Embase | ('factor XI inhibitor'/exp OR 'factor XIa inhibitor' OR 'Abelacimab' OR 'Milvexian' OR 'Asundexian' OR 'Osocimab') AND ('atrial fibrillation'/exp OR 'AF' OR 'non-valvular atrial fibrillation' OR 'NVAF') AND ('stroke prevention' OR 'thromboembolism prevention' OR 'cardioembolic stroke') AND ('anticoagulation'/exp OR 'anticoagulant'/exp OR 'direct oral anticoagulant' OR 'DOAC' OR 'NOAC' OR 'warfarin'/exp OR 'vitamin K antagonist') | 29 |

DOAC: Direct Oral Anticoagulants, NVAF: Non-valvular atrial fibrillation, NOAC: Non Vitamin K anticoagulant, VKA: Vitamin K Antagonist

Supplemental table 2.Summary of Findings Table (GRADE Assessment)

| **Outcomes** | **Risk with Factor XI/XI A inhibitor** | **Risk with DOAC** | **Relative effect (95% CI)** | **No of participants (studies)** | **Certainty of the evidence (GRADE)** | **Comments** |
| --- | --- | --- | --- | --- | --- | --- |
| All-Cause Mortality | 17,460 | 16,762 | RR 0.82 (0.77–0.88) | 3 studies | ⊕ ⊕ ⊕ ⊕ High | No concerns; precise and consistent |
| Major Bleeding | 17,460 | 16,762 | RR 0.41 (0.36–0.46) | 3 studies | ⊕ ⊕ ⊕ ⊕ High | No concerns; strong evidence of benefit |
| Ischemic Stroke | 17,460 | 16,762 | RR 3.42 (2.62–4.46) | 3 studies | ⊕ ⊕ ⊕ ⊕ High | Elevated risk with intervention; consistent |
| Hemorrhagic Stroke | 8,225 | 7,792 | RR 0.14 (0.01–2.85) | 2 studies | ⊕ ⊕ ○○ Low | Very serious imprecision; few events |
| Systemic Embolism | 8,730 | 8,042 | RR 4.26 (0.63–28.73) | 3 studies | ⊕ ⊕ ○○ Low | Very serious imprecision; wide CI |
| Death from Cardiac Cause | 7,878 | 7,614 | RR 1.05 (0.21–5.33) | 3 studies | ⊕ ⊕ ⊕ ○ Moderate | Serious imprecision; wide confidence interval |
| Serious Adverse Events | 1,357 | 678 | RR 0.95 (0.70–1.31) | 3 studies | ⊕ ⊕ ⊕ ○ Moderate | Serious imprecision; not statistically significant |

**Supplemental figure1. Dose-based subgroup analysis for the outcome of major bleeding**

**
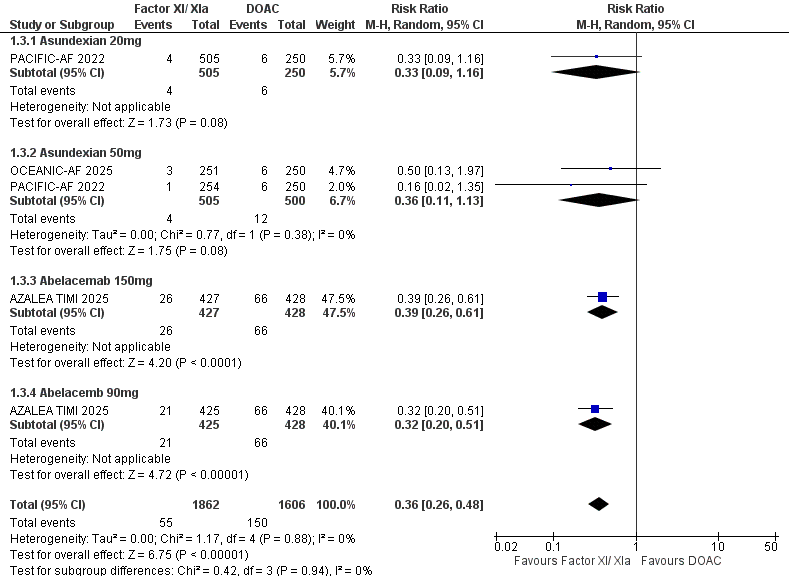
**

**Supplemental figure2. Dose-based subgroup analysis for the outcome of ischemic stroke**

**
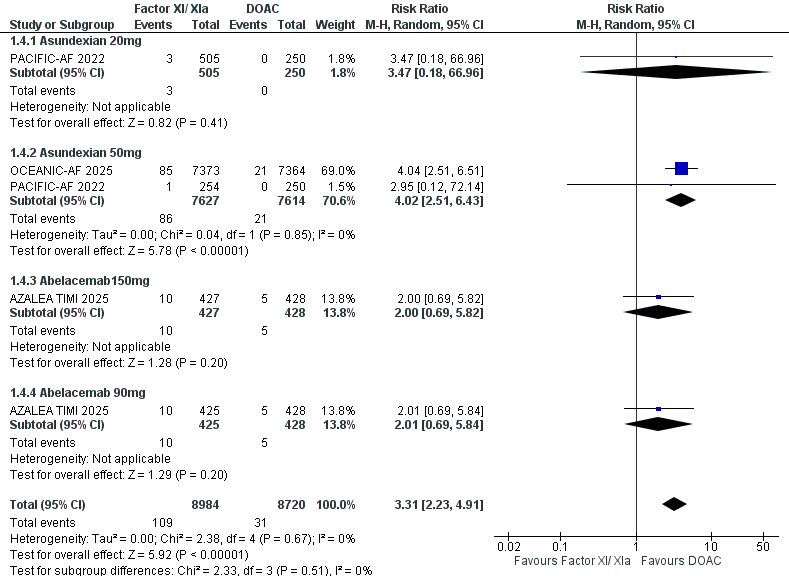
**

**Supplemental figure3. Dose-based subgroup analysis for the outcome of all-cause mortality
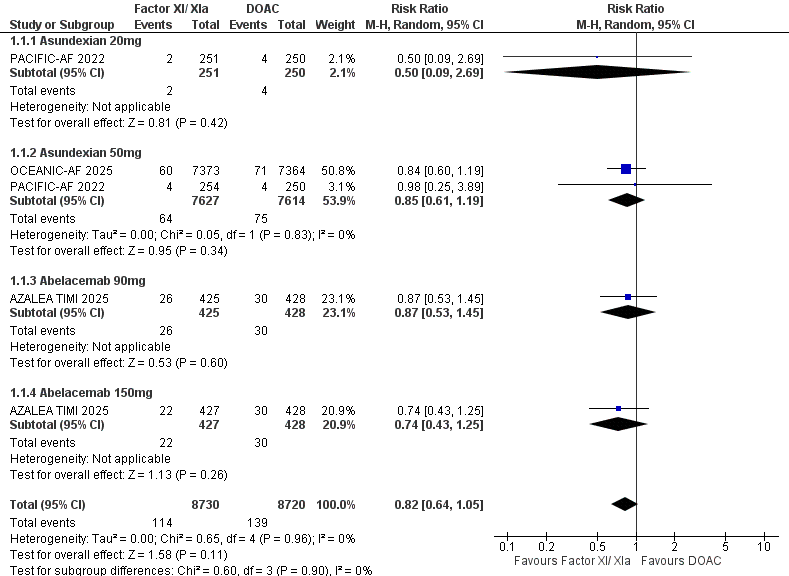
**

**Supplemental figure4. Trial Sequential Analysis (TSA) assessing the effect of Factor XI/XIa inhibitors verses DOAC on the outcomes of (A)Major Bleeding (B)Ischemic stroke (C)Haemorrhagic stroke (D)Systemic embolism (E)All-cause mortality (E)Cardiovascular mortality (F)Serious Adverse Events (A)**,m **
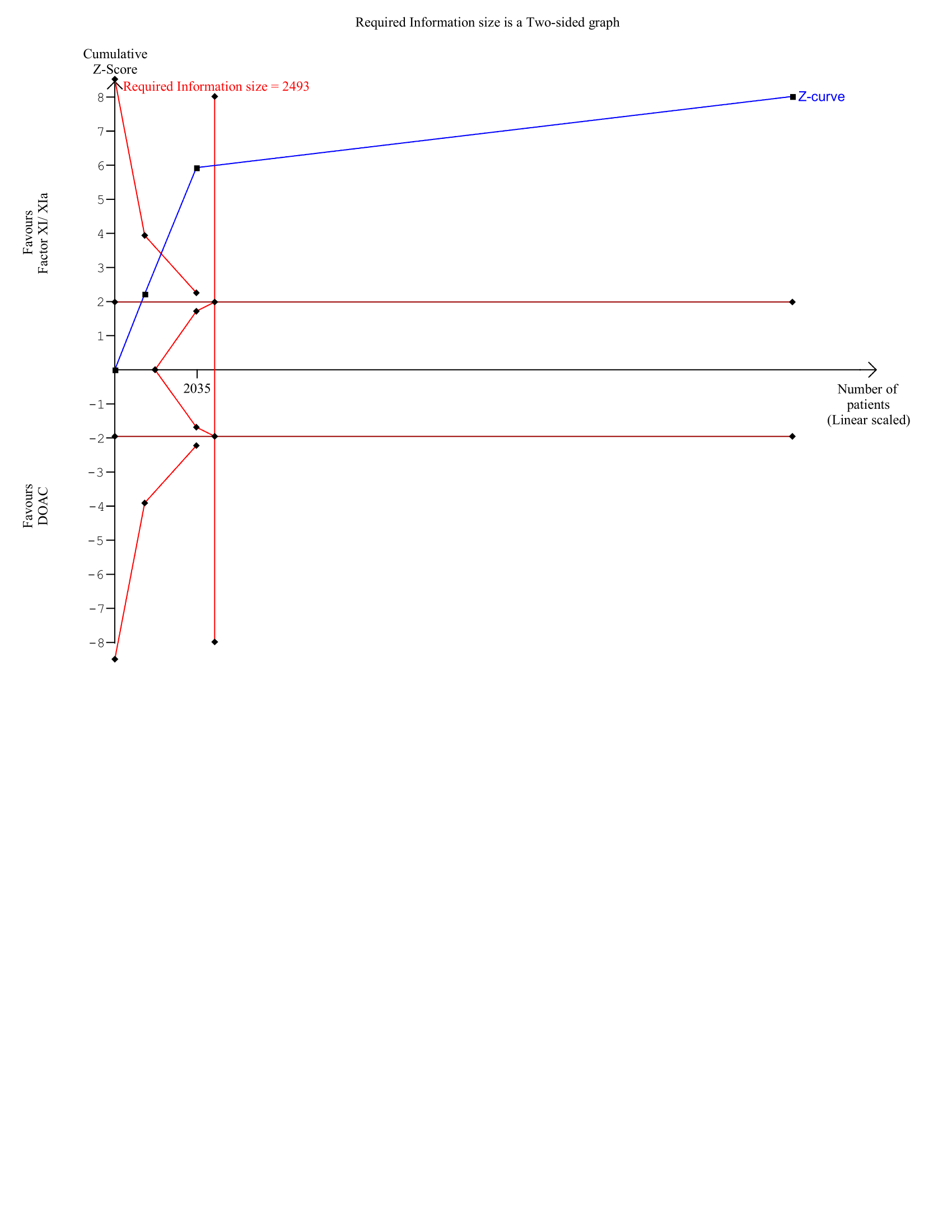
**

**(B)** **
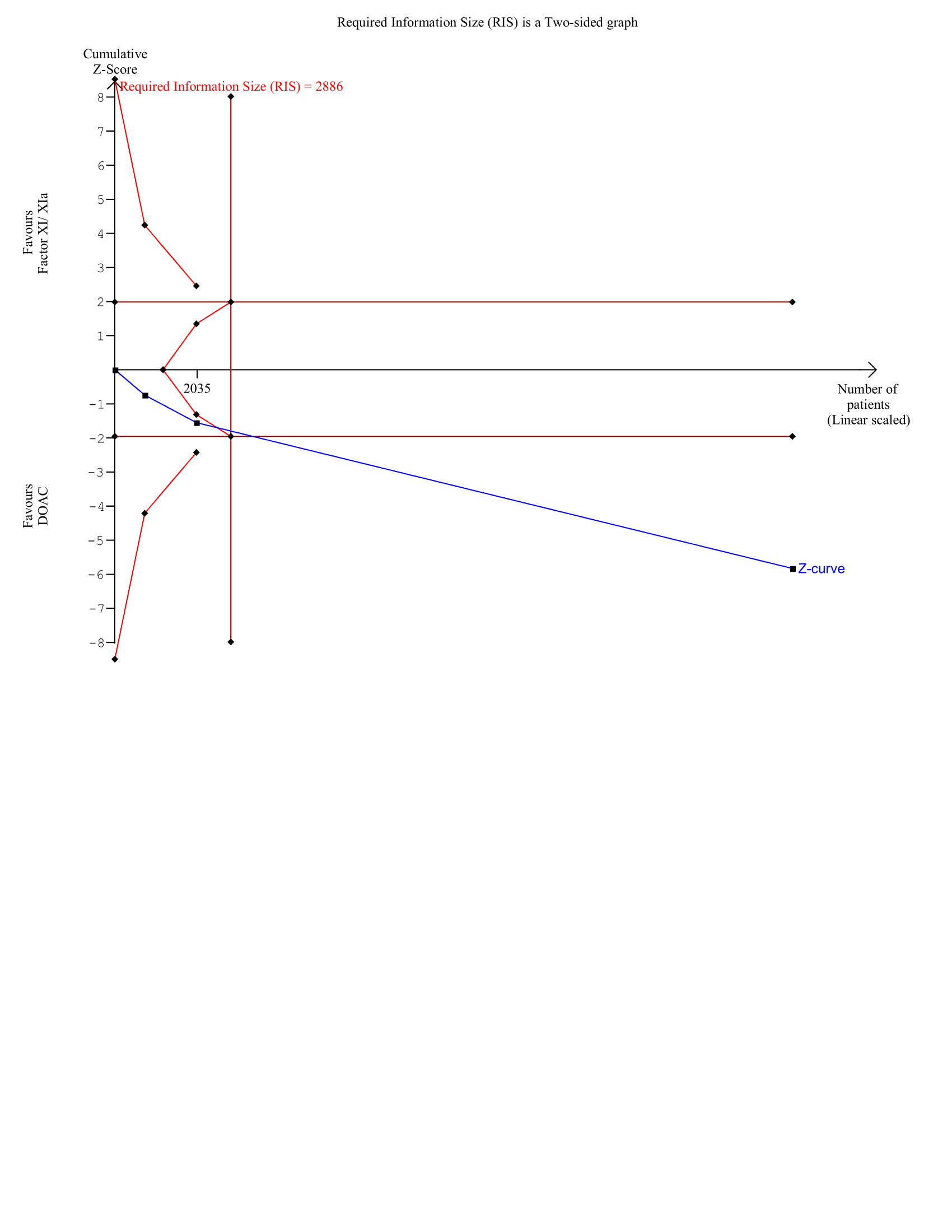
**

**(C)** **
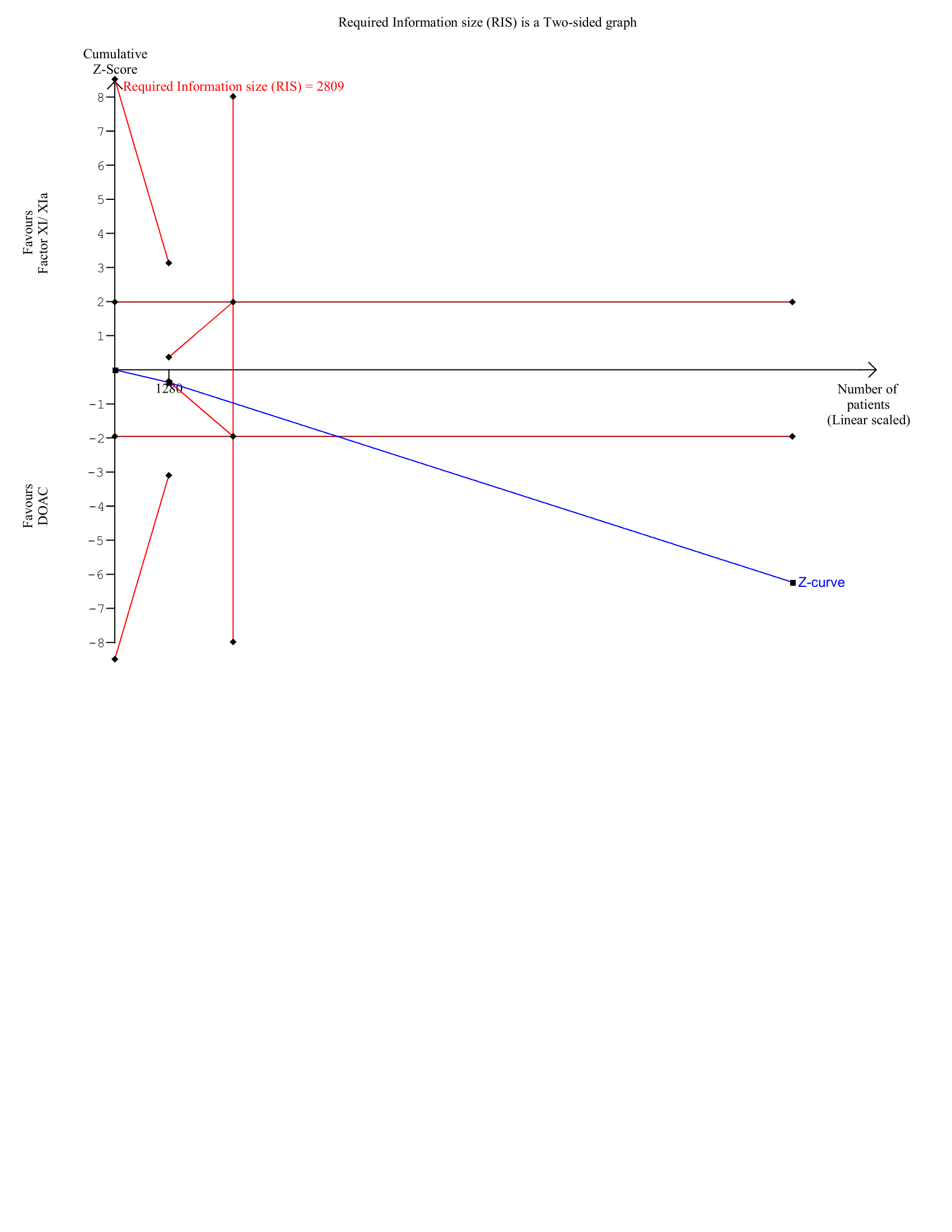
(D)
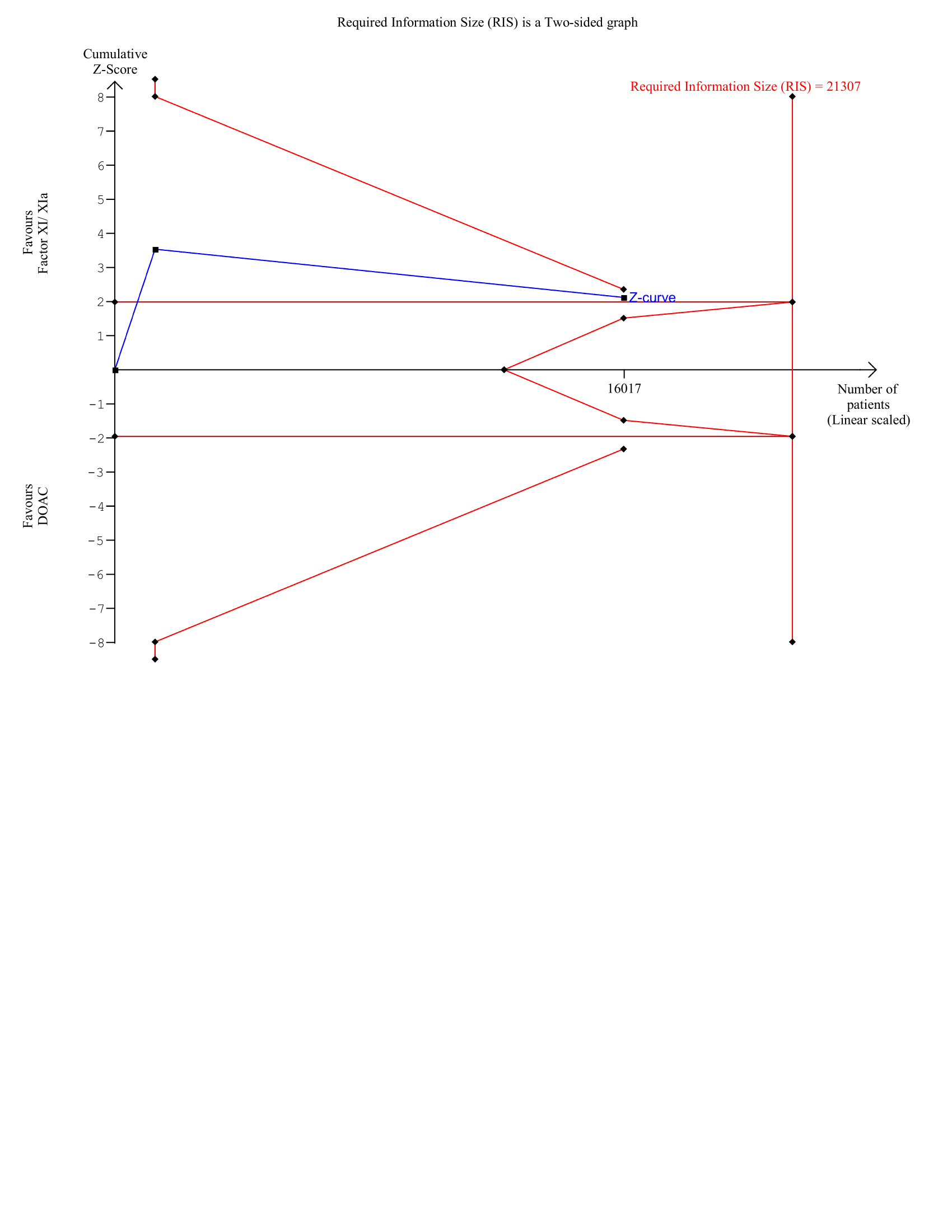
**

**(E)**

**
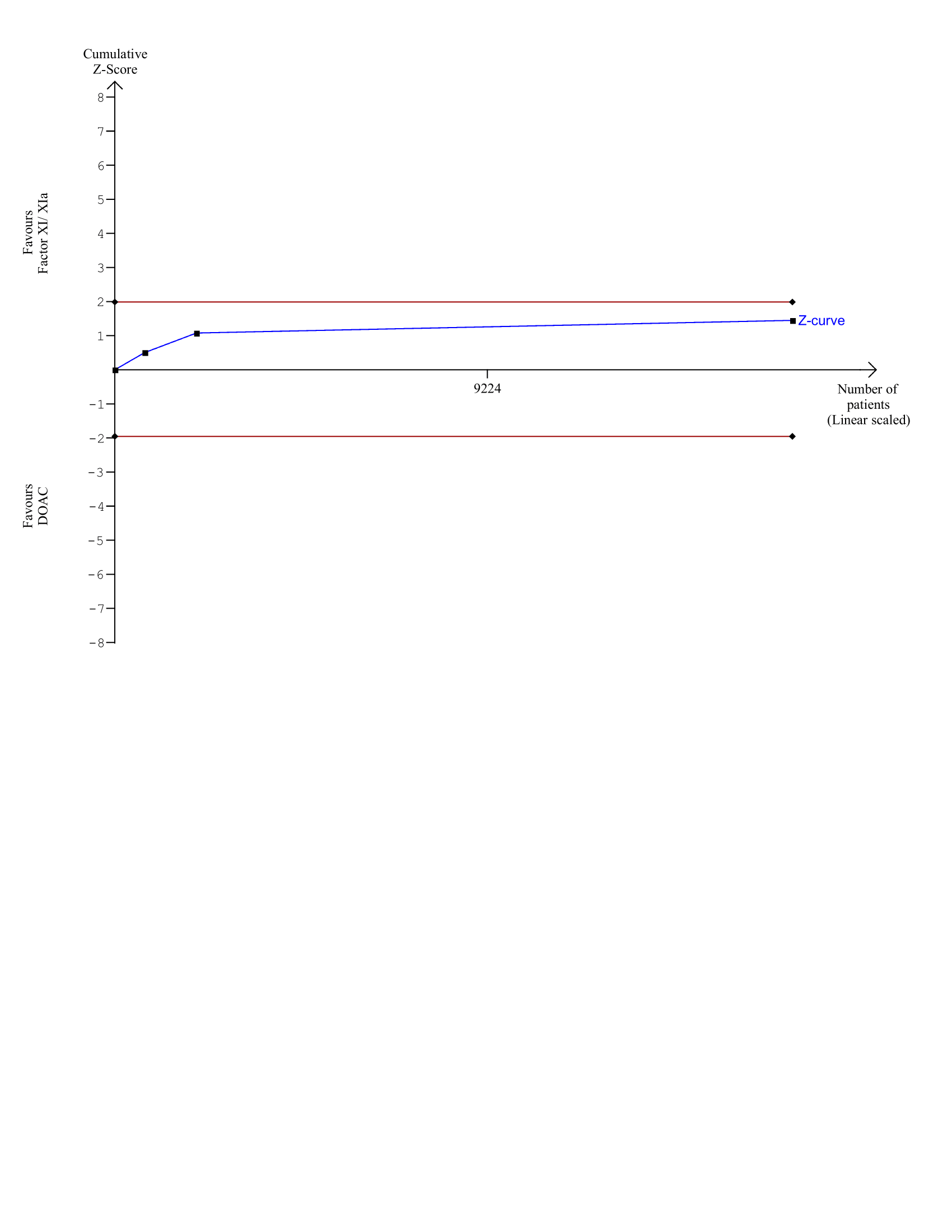
**

**(F)** **
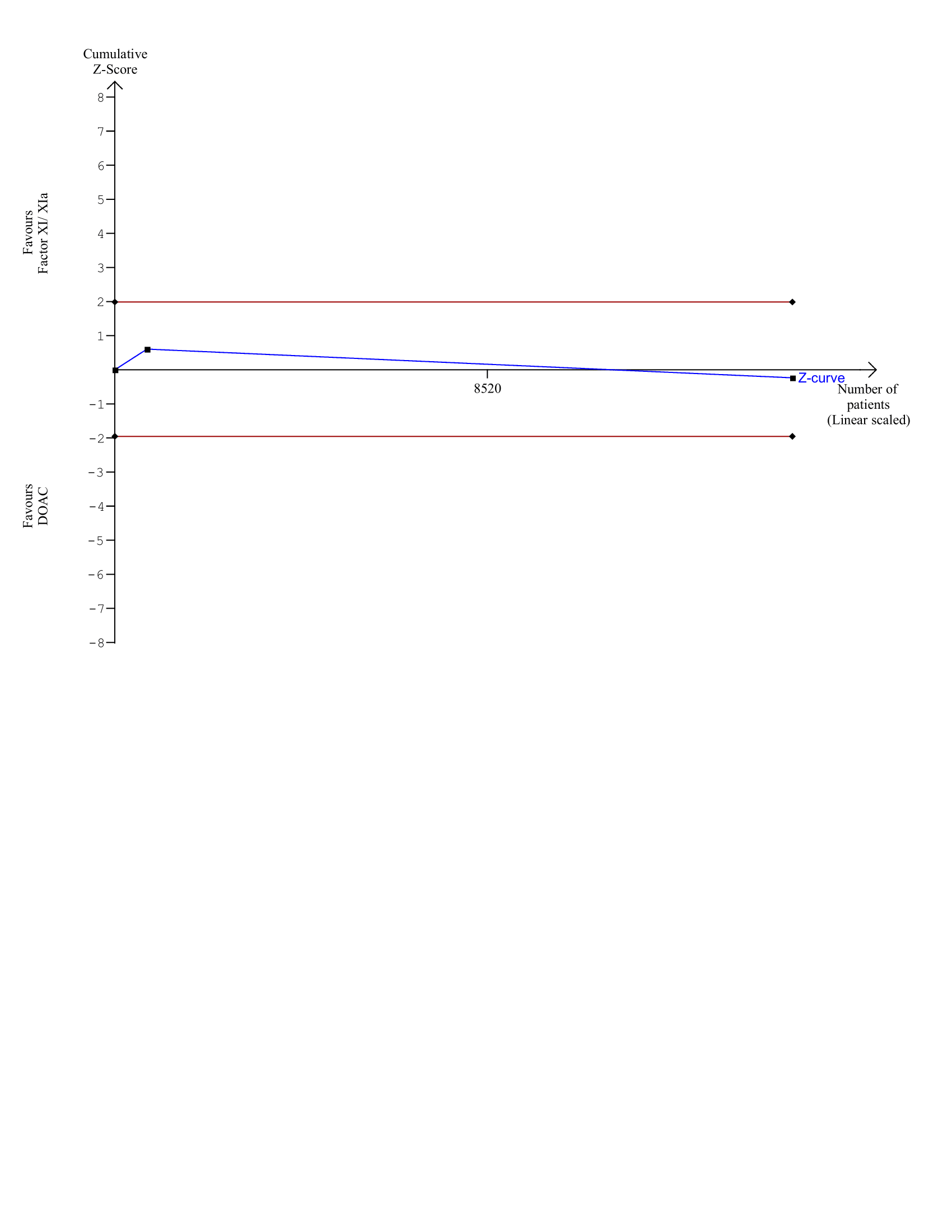
**

**(G)** **
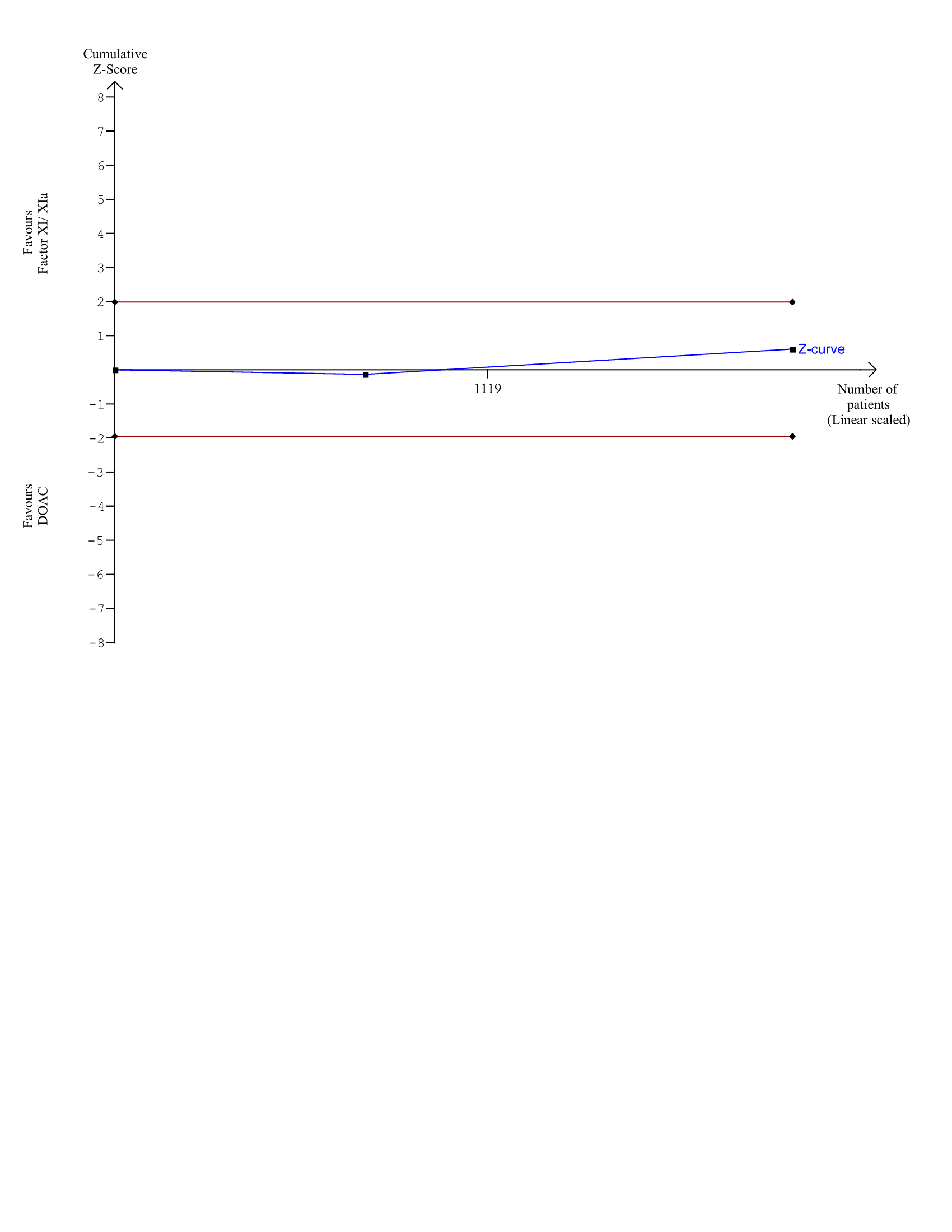
**

**Supplemental figure5.**Risk of bias summary


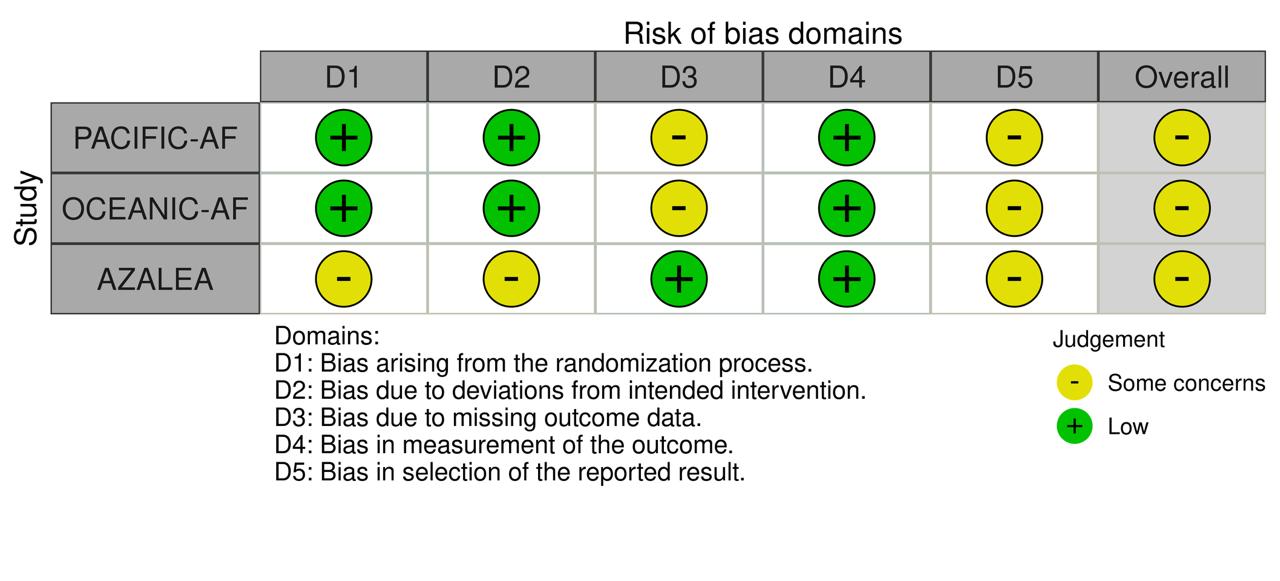


**Supplemental figure6.**Risk of bias graph


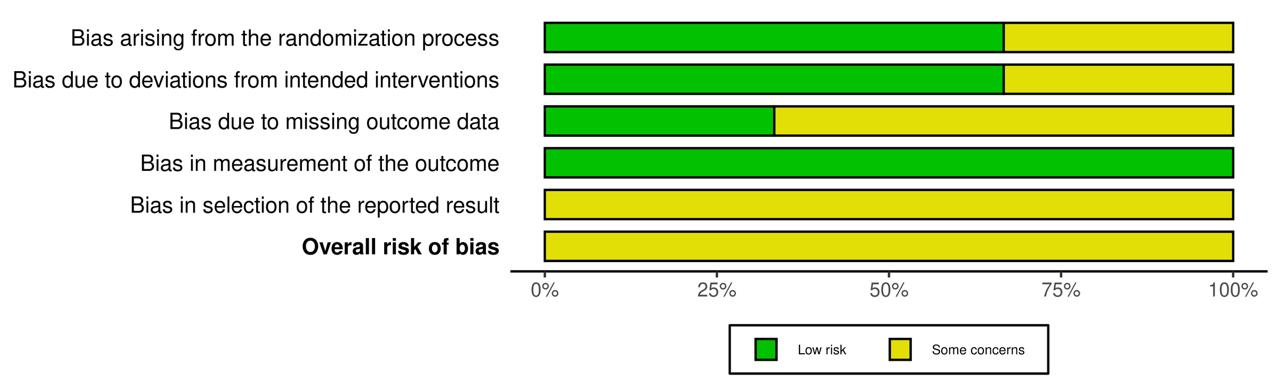

Supplement: Supplementary file 1 — Supplemental Figure 1: Dose‐based subgroup analysis for the outcome of major bleeding. Supplemental Figure 2: Dose‐based subgroup analysis for the outcome of ischemic stroke. Supplemental Figure 3: Dose‐based subgroup analysis for the outcome of all‐cause mortality. Supplemental Figure 4: Trial Sequential Analysis (TSA) assessing the effect of Factor XI/XIa inhibitors verses DOAC on the outcomes of (A) Major Bleeding (B) Ischemic stroke (C) Haemorrhagic stroke (D) Systemic embolism (E) All‐cause mortality (E) Cardiovascular mortality (F) Serious Adverse Events. Supplemental Figure 5: Risk of bias summary. Supplemental Figure 6: Risk of bias graph. Supplemental Table 1: Detailed search strategy across different data bases. Supplemental Table 2: Summary of Findings Table (GRADE Assessment). [file CLC-49-e70263-s001.docx]
